# China's new ethical guidelines for the use of brain–computer interfaces

**DOI:** 10.1093/nsr/nwae154

**Published:** 2024-05-10

**Authors:** Mu-ming Poo

**Affiliations:** Scientific Director, Institute of Neuroscience, CAS Center for Excellence in Brain Science and Intelligence Technology, China

While the world is bewildered by the dazzling advances of generative artificial intelligence and struggling to find consensual guidelines for its governance, the rapid pace of research on the brain–computer interface (BCI) also calls for governance on its development and application. In early February, China's Ministry of Science and Technology (MoST) announced the ‘Guidelines for Research Ethics in Brain-computer Interface’ [1] based on recommendations of the National Research Ethics Committee. Its content is largely in line with an earlier announcement in October 2023 by the Psychiatry Society of China [2] on ‘Ethics Governance of Brain-Computer Interface Research in Mental Disorders’. Both guidelines are formulated on the principles of the World Medical Association Declaration of Helsinki on ‘Ethical Principles for Medical Research Involving Human Subjects’ in 2008.

The goal of both guidelines is to clarify the ethical norms for BCI research and application, and to promote the sustainable development of BCI technology that enables monitoring, decoding and manipulating human brain activity. Invasive BCI involves putting biocompatible recording or stimulation electrodes (i.e. interfaces) into brain tissue at varying depths. Non-invasive approaches enable recording or stimulating brain activity from outside the brain by putting interfaces on the scalp. Machine-learning algorithms are often used to help in decoding the cortical signals of the brain for controlling external devices such as neuroprosthetic arms. In the opposite direction, signals generated by external devices could be used to stimulate brain activity that may restore impaired brain functions, as exemplified by deep brain stimulation (DBS) in severe Parkinson's disease patients to regain mobility. Active research is ongoing for the use of DBS or non-invasive stimulation for treating major depressive and other brain disorders.

The guidelines announced by the Psychiatry Society of China [2] are extensive in coverage and depth. Participating experts involved in formulating these guidelines also came from diverse disciplines, including neuroscience, psychiatry, neurosurgery, medical engineering, computer and information science, ethics, philosophy of science and technology, psychology and law. Six proposed criteria for ethical practices of BCI-related research and therapeutic application are briefly summarized below.

- Scientific soundness—fully consider available scientific evidence, have scientifically sound and rigorous research protocols, ensure benefit to the subject and, in the absence of expected benefit to the subject, ensure sufficient social benefit with minimal risk to the human subject.
- Beneficence—yield the maximal benefit in the diagnosis, intervention or rehabilitation of brain disorders, under the consideration of risk/benefit balance and the overall consideration of the benefit to the individual and general public.
- Autonomy—protect the right to know and the decision of the subject or guardian, strictly follow the right-to-know procedure without deception, inducement or coercion and allow the subject or guardian to withdraw at any stage of the study.
- Minimum harm—assess the short- and long-term benefits for the subject and control potential risk such as the biocompatibility of the implanted material, with non-invasive overriding invasive approaches and personal safety/health benefits overriding scientific/social benefit considerations.
- Privacy—establish normalized regulations for data collection, technical standards for anonymization and de-identification, including general information and data on brain structure and functional activity.
- Fairness and justice—adhere to unbiased selection, enrollment and exclusion of treatment subjects and distribution of research benefit, risk and burden.

As BCI technology further develops and becomes widely applied, a more difficult ethical issue is its use for augmenting normal brain functions rather than for therapeutic purposes, applications such as non-invasive neuromodulation of sleep/wakefulness, attention, emotion, as well as learning and memory functions. The MoST guidelines include specific requirements from this aspect, including thorough risk–benefit assessment and more strict control of its use, in order to minimize addiction, adverse effects on human reasoning, decision-making and cognitive behaviors, and negative impacts on social fairness and individual autonomy.

We are only at the beginning of understanding how the human brain works, how to decode brain signals, what goes wrong in various brain disorders and what will be the consequence of manipulating brain activity. In fact, most BCI applications for therapeutic purposes are currently practiced without solid scientific basis in terms of understanding the most relevant brain targets and the long-term effects of BCI on the brain. Advances in BCI technology thus need to be aligned better with our understanding of the brain. It is often cutting-edge progress in frontier areas that calls for new regulatory guidelines and the updating of guidelines is required as the frontier expands. In the case of BCI, advances in basic brain science must play an important role in setting future guidelines for BCI. We look forward to evolving new guidelines that help to shape BCI development towards the ever-changing needs of our society.

***Conflict of interest statement*.** None declared.
